# *Trachymolgus purpureus* sp. n., an armored snout mite (Acari, Bdellidae) from the Ozark highlands: morphology, development, and key to *Trachymolgus* Berlese

**DOI:** 10.3897/zookeys.125.1875

**Published:** 2011-08-26

**Authors:** J. Ray Fisher, Michael J. Skvarla, Gary R. Bauchan, Ronald Ochoa, Ashley P.G. Dowling

**Affiliations:** 1 Department of Entomology, 319 Agriculture Bldg., Fayetteville, AR, 72701, USA; 2 USDA, ARS, Electron and Confocal Microscopy Unit, Beltsville, MD, 20705; 3 USDA, ARS, Systematic Entomology Laboratory, Beltsville, MD, 20705

**Keywords:** LT-SEM, taxonomy, Prostigmata, new species, image diversity

## Abstract

*Trachymolgus purpureus* Fisher & Dowling **sp. n.** is described from the Ozark highlands of North America. A diversity of imaging techniques are used to illustrate the species including low-temperature scanning electron microscopy (LT-SEM), stereomicrography, compound light micrography, and digitally created line drawings. Developmental stages (larva, nymphs, and adult) and morphology are illustrated and discussed, and terminological corrections are suggested. *Trachymolgus recki* Gomelauri, 1961 is regarded as being described from tritonymphs. A key to *Trachymolgus* is presented.

## Introduction

**Figure 1.**
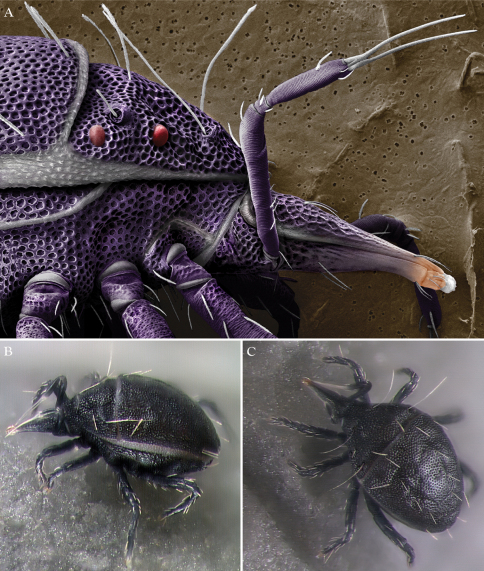
*Trachymolgus purpureus* **sp. n.** **A** Lateral view of proterosoma, LT-SEM; **B–C** Stereomicrographs of live specimens.

**Figure 2.**
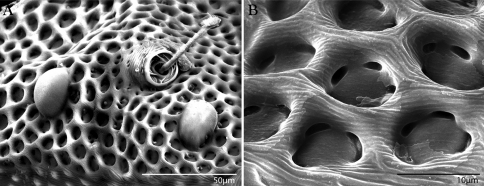
Integument of *Trachymolgus purpureus* sp. n. LT-SEM. **A** Lateral view of eyes and *pt* showing foveolate indentions **B** Magnified view of foveolae and pits.

Bdellidae Dugès, 1834 generally have a striated, unsclerotized integument. Exceptions occur in Cytinae Grandjean, 1938, which comprises three of the most distinctive bdelloid genera. *Cyta* Heyden, 1826 are common mites known for their stocky bodies, massive chelicerae, and unpaired fifth eye. *Rigibdella ignea* Tseng, 1978 from Taiwan have sclerotized, striated holodorsal shields (Tseng 1978; Lin and Zhang 2010). The only other bdellids with a sclerotized body are the subject of this study – *Trachymolgus* Berlese, 1923. *Trachymolgus* are infrequently collected mites with three described species. Instead of the typical fingerprint-like membranes of other Bdellidae, the integument of *Trachymolgus* is heavily sclerotized, divided into distinct sclerites, and dark (Fig. 1). The sclerites are foveolate, containing many indentions that create a characteristic reticulated patterning (Fig. 2a). These indentions have multiple small pits at the corners of the foveolae (Fig. 2b). This strange integument, combined with the rarity of collection, has rendered *Trachymolgus* the most enigmatic bdellid.

Berlese (1923) erected *Trachymolgus* to accommodate the distinctive snout mite Canestrini and Fanzago (1876) named *Bdella nigerrima*. *Trachymolgus niggerimus* (Canestrini & Fanzago, 1876) was originally collected in northern Italy (Padova) and subsequently reported from Sicily and Lugano, Switzerland (Thor 1931), Crimea, Ukraine (Vainshtein et al. 1978), Caucasus (Vainshtein et al. 1978), and from rodent nests in Bulgaria (Sosnina et al. 1965). Grandjean (1938) grouped *Cyta* and *Trachymolgus* into a new subfamily (Cytinae) based on the number of subcapitular setae and leg trichobothria, presence of pseudotracheae, and normal chelicerae (reduced in Spinibdellinae). Two other species were described later, both known only from their type localities: *Trachymolgus recki* Gomelauri, 1961 in Georgia (former U.S.S.R.: Tbilisi and Kashtak) and *Trachymolgus jesusi* Mejia-Recamier & Palacios-Vargas, 1999 in Mexico (Jalisco and Chamela). Undetermined species have been reported from a Buddhist temple on Shikoku Island, Japan (Nakamura et al. 2006), from the St. Lawrence Islands National Park, Canada (Smith et al. 1996), and we collected a third from Columbus, Ohio. Here, we describe and illustrate *Trachymolgus purpureus* sp. n. from the Ozark highlands of North America.

## Material and methods

### Specimens

Mites were collected primarily from leaf litter samples in the Ozark Mountains of Arkansas (U.S.A.), specifically Buffalo National River and Devil’s Den State Park, and extracted using Berlese-Tullgren funnels. Approximately half of the specimens were slide-mounted with Hoyer’s medium (see Krantz and Walter 2009 for preparation), and half are stored in 95% ethanol at -80°C. Slide-mounted specimens were dissected along the frontal plane. Five paratypes are deposited in the Ohio State University Acarology Collection, Columbus, Ohio. Four paratypes each are deposited in the Field Museum of Natural History, Chicago, Ill., and the National Mite Collection, National Museum of Natural History, Smithsonian Institution, located in Beltsville, Md. All other type specimens are deposited in the Acari Collection of the University of Arkansas, Fayetteville, Ark.

### Terminology

An effort is made to implement terminology that is broadly applicable and well accepted across acariforms despite conventions used among bdelloid authors. Thus, two terms have been renamed herein. First, “hypostome” is used by many (Atyeo 1960; van der Schyff et al. 2004; Hernandes and Feres 2006; Krantz and Walter 2009) to refer to the entire subcapitulum, though it more accurately refers only to the portion anterior to the oral opening (Evans 1992; Krantz and Walter 2009). Thus, in addition to the subcapitulum itself, its setae have been renamed herein to ventral subcapitular setae (*vs*) and dorsal subcapitular setae (*ds*).

Second, the major idiosomal divisions of bdelloids are regularly referred to as the “propodosoma” and “hysterosoma” (Bdellidae: Atyeo 1960, Mejia-Recamier and Palacios-Vargas 1999, Hernandes et al. 2007; Cunaxidae: Meyer and Ryke 1959, Swift 1996, Den Heyer and Castro 2009). However, acariform segmentation is a debated topic with recent views favoring a radically reduced podosoma leaving the major idiosomal divisions (when viewed dorsally) the opisthosoma and aspidosoma (Grandjean 1969; Coineau 1974; Evans 1992; Krantz and Walter 2009). With regard to the posterior portion, hysterosoma is already widely used and accurately refers to the idiosoma posterior to the sejugal furrow (metapodosoma and opisthosoma). Moreover, it is independent of segmentation hypotheses. Therefore, we retain the use of hysterosoma. However, propodosoma is hypothesis dependent. The Grandjean (1969) hypothesis of body organization suggests the propodosoma is highly reduced dorsally, rendering propodosoma inaccurate when referring to the anteriodorsal portion of the idiosoma. Instead, aspidosoma accurately describes this region. Therefore, we abandon the use of propodosoma when referring to the dorsal morphology of acariforms. However, until more evidence exists for segmentation homologies, we also avoid the use of aspidosoma for Bdelloidea. Prodorsum is widely used by acarologists, but is usually poorly defined and represents the opposite extreme from aspidosoma--straying too far from morphological hypotheses. An exception to this is in Oribatida (e.g., Sanders and Norton 2004), where it is often used as synonymous with the aspidosomal shield (=aspis) and is therefore hypothesis dependent. Thus we recommend restricting prodorsum to casual use. Proterosoma refers to the body anterior to the sejugal furrow (propodosoma, aspidosoma, and gnathosoma of Grandjean [1969]), and is a segmentation independent term that is already widely used in acarology. Therefore, we adopt proterosoma here.

With regard to hysterosomal setal notation, we follow the chaetotaxic system of (Grandjean (1939, 1947) that has been widely adopted (e.g., van der Hammen 1970; Lindquist 1976, 1977; Kethley 1990). Proterosomal setal notation in this system is currently problematic. Generally, we do not recommend the use of mixed approaches in terminology, especially given our attempts to utilize broadly applicable notations. However, recent suggestions in the proterosomal setal notation of bdelloids have rendered this system unreliable. In the Grandjean system, proterosomal setae are termed internal/external verticals (*vi* and *ve*) and internal/external scapulars (*sci* and *sce*). In Bdelloidea, *sci* are always external to *sce*, leaving the internal/external designations unintuitive. Den Heyer and Castro (2008a) noted this and proposed simply switching the terms when referring to bdelloids so that *sce* and *sci* are descriptive, which they have published since (Den Heyer and Castro 2008b, c; Den Heyer 2011). As a result, the literature now has *sce* and *sci* referring to both inner and outer scapulars. The homology of these setae with respect to other mites is not known. Therefore, until more evidence is provided for homology, we resort to a modified version of Atyeo (1960) when referring to proterosomal setae, which unambiguously relies on position: anterior and posterior trichobothria (*at* and *pt*), and lateral and median proterosomal setae (*lps* and *mps*).

Leg chaetotaxy follows Grandjean’s system as reviewed by Norton (1977). However, leg chaetotaxy is poorly studied among Eupodina, and only distal tarsal setae are denoted presently, which has been adopted by other eupodine authors (e.g., Jesionowska 2010). Nevertheless, we believe Grandjean’s system can be employed with other leg setae, and will readdress this in a more detailed forthcoming study.

### Images

Most species descriptions include only a few image types; line drawings are most common in acarology (e.g., Mejia-Recamier and Palacios-Vargas 1999; Den Heyer and Castro 2008a, b, c; Łochyńska 2008). We believe image diversity increases accuracy, accessibility, and attractiveness of taxonomic products, and have therefore included many images and a range of imaging types. Other acarologists have also begun including image diversity in taxonomic works (e.g. Mąkol 2010; Wohltmann 2010; Pešić et al. 2011).

Line drawings were created digitally with Adobe Illustrator CS5 and a Wacom Cintiq 21UX tablet using procedures outlined in Fisher and Dowling (2010). Compound light micrographs were taken with a Leica DFC300 FX camera and a Leica DM2500 DIC light microscope. Stereomicrographs were taken with a Leica MZ 16 stereoscope and a Leica DFC 290 camera. Photographs were enhanced using Adobe® Photoshop CS4.

Low-temperature scanning electron micrographs (LT-SEM) were made using an S-4700 field emission scanning electron microscope (Hitachi High Technologies America, Inc., Pleasanton, Calif.) equipped with a Quorum CryoPrep PP2000 (Energy Bean Sciences, East Grandby, Conn.) cryotransfer system. To prepare specimens, mites were placed on 12 mm diameter ultra smooth carbon double sided adhesive tabs (Electron Microscopy Sciences, Hatfield, PA) which were adhered to flat specimen holders consisting of 16x30mm copper plates that were tacked on the edges to the tabs with a small dot of Tissue Tek (OCT Compound, Ted Pella, Inc., Redding, Calif.), which acted as the cyro-adhesive upon freezing. The samples were frozen conductively, in a Styrofoam box, by placing the plates on the surface of a pre-cooled (-96°C) brass bar whose lower half was submerged in liquid nitrogen (LN2). After 20–30s, the holders containing the frozen samples were transferred to a LN2 Dewar for future use or cryotransferred under vacuum to the cold stage in the pre-chamber of the cryotransfer system. Removal of any surface contamination (condensed water vapor) took place in the cryotransfer system by etching the frozen specimens for 10–15 min by raising the temperature of the stage to -90°C. Following etching, the temperature was lowered below -130°C, and a magnetron sputter head equipped with a platinum target, was used to coat the specimens with a very fine layer of platinum. The specimens were transferred to a pre-cooled (-130°C) cryostage in the SEM for observation. An accelerating voltage of 5kV was used to view the specimens. Images were captured using a 4pi Analysis system (Durham, N.C.). Images were sized and placed together into figures using Adobe® Photoshop 7.0 and CS4.

## Taxonomy

### Trachymolgus purpureus

Fisher & Dowling sp. n.

urn:lsid:zoobank.org:act:E0FAE922-2B81-4FB5-8D50-CE0F93517CD2

http://species-id.net/wiki/Trachymolgus_purpureus

#### Diagnosis.

*Trachymolgus purpureus* sp. n. is heavily armored with distinctive integument characteristic of *Trachymolgus* (Figs 1–2). Like *Trachymolgus jesusi*, the integument is dark purple, whereas *Trachymolgus nigerrimus* was described as black. Like *Trachymolgus jesusi* and *Trachymolgus nigerrimus*, there are two teeth on the fixed cheliceral digit. Like *Trachymolgus nigerrimus*, *Trachymolgus purpureus* has one tooth on the movable digit (*Trachymolgus jesusi* have three) and a serrated edge proximal to the tooth (undescribed in other species). All stages have two pairs of eyes, unlike the larva, proto- and deutonymphs of *Trachymolgus jesusi*, which lack eyes (tritonymphs and adults have two pairs). *Trachymolgus purpureus* pedipalpal basi- and telofemora are only fused dorsally. *Trachymolgus jesusi* pedipalp femora are completely fused, whereas *Trachymolgus nigerrimus* are completely divided. *Trachymolgus purpureus*, like other *Trachymolgus*,have undivided femora on legs I-II (femora III-IV are divided). All other Bdellidae have divided femora on all legs. *Trachymolgus jesusi* is the only bdellid reported to have undivided femora on legs II and III. The ontogeny of *Trachymolgus purpureus* differs markedly from that described for *Trachymolgus jesusi*, the only other species where ontogeny was investigated. Finally, there are many chaetotaxic differences on the appendages and venter between *Trachymolgus purpureus* and *Trachymolgus jesusi*. Most chaetotaxy of *Trachymolgus nigerrimus* remain to be investigated. See Remarks for discussion of *Trachymolgus recki*.

##### Imago description.

Females and males similar, except for genitalia, size, and chaetotaxic differences noted in Table 1. Color dark purple; occasionally immatures and adults were collected from the Buffalo National River (Arkansas) with an internally green coloration, which rendered the normally purple mite teal; teal specimens returned to purple after a few days in 95% ethanol, and were indistinguishable from normal specimens when slide-mounted (we also collected *Penthaleus*, a normally black to dark blue mite, from the same habitat exhibiting green internal coloration). Integument divided into heavily armored sclerites with foveolate sculpturing (Fig. 2a). The foveolate indentions (foveolae) are bordered with pits (Fig. 2b). Measurements in Tables 2–5.

**Table 1.** Leg chaetotaxy. Female (♀), male (♂), tritonymph (3N), deutonymph (2N), protonymph (1N), larva (L), pedipalp (Pp), legs I-IV (I-IV). Numbers represent setal counts for barbulate setae (undesignated), solenidia (s), and trichobothria (tr). Male setal counts that are not different from the female are denoted with an asterisk (*). Absent characters are denoted with a dash (-). Fused segments are denoted by fused cells. Numbers in parentheses denote occurrences of two solenidia on tarsus II in some specimens.

Stage		Coxa	Trochanter	Basifemur	Telofemur	Genu	Tibia	Tarsus
♀	Pp	-	0	8		4	4; 1s	
	I	7	2	18		4; 2s	8; 2s; 1tr	28; 5s
	II	7	2	20		4; 1s	9; 1s	26; 1(2)s
	III	9	2	10	10	5; 1s	9; 1s	24; 1tr
	IV	8	2	10	10	5; 1s	9; 1tr	21; 1s
♂	Pp	-	*	*		*	*	
	I	6	*	*		*	*	*
	II	6	*	*		*	*	*
	III	6	*	*	*	*	*	*
	IV	10	*	*	*	*	*	*
3 N	Pp	-	0	6		4	4; 1s	
	I	4	2	18		4; 2s	8; 2s; 1tr	24; 5s
	II	4	2	18		4; 1s	8; 1s	22; 1s
	III	4	2	9	9	4; 1s	9; 1s	20; 1tr
	IV	3	1	6	7	4; 1s	8; 1tr	19; 1s
2 N	Pp	-	0	4-5		4	4; 1s	
	I	4	1	12		4; 2s	7; 2s; 1tr	20; 5s
	II	2	1	11		4; 1s	6; 1s	18; 1(2)s
	III	4	1	6	6	4; 1s	6; 1s	16; 1tr
	IV	2	1	2	4	4; 1s	6; 1tr	15; 1s
1 N	Pp	-	0	4		4	4; 1s	
	I	2	1	7		4; 2s	5; 2s	18; 4s
	II	1	1	6		4; 1s	5; 1s	16; 1s
	III	1	1	1	4	4; 1s	5; 1s	12; 1tr
	IV	0	0	0		0	1	7
L	Pp	-	0	2		4	4; 1s	
	I	3	0	7		4; 2s	5; 2s	16; 3s
	II	1	0	6		4; 1s	5; 1s	14; 1s
	III	2	0	6		4; 1s	5; 1s	12; 1tr
	IV	-	-	-		-	-	-

**Table 2.** Body measurements. Stage (St), female (♀), male (♂), tritonymph (3N), deutonymph (2N), protonymph (1N), and larva (L), mean (M), standard deviation (S), range (R), number examined (n), idiosomal length (Idi L) and width (Idi W), and lengths of proterosomal shield (Pro), hysterosomal shield (Hys), lateral shield (Lat), subcapitulum (Sub), chelicerae (Chel), pedipalps (Ped), anal shield (Ana), genital shield (Gen), and legs I-IV (L I-IV). Absent characters are denoted with a dash (-). All measurements in micrometers.

St		Idi L	Idi W	Pro	Hys	Lat	Sub	Chel	Ped	Ana	Gen	I	II	III	IV
♀	M	791	505	279	512	485	307	283	344	113	171	470	457	526	600
	S	24	14	9	16	11	13	7	4	5	9	9	13	6	37
	R	768-838	488-525	273-298	495-540	475-503	285-323	273-295	338-350	108-120	160-190	458-483	438-478	515-533	538-665
	n	7	7	7	7	7	8	8	6	7	8	7	7	7	8
♂	M	753	483	270	483	448	294	277	345	105	165	468	445	518	594
	S	52	25	10	45	34	8	9	7	8	9	16	13	21	25
	R	693-825	465-500	260-283	425-545	390-475	288-305	268-293	338-355	95-118	150-175	443-483	435-465	488-540	555-628
	n	6	2	6	6	6	5	6	5	6	6	6	6	6	6
3N	M	684	452	259	268	134	258	242	301	95	102	393	378	451	509
	S	99	70	38	52	10	10	10	12	6	4	23	27	17	43
	R	588-808	350-500	230-314	220-324	126-145	250-273	228-255	288-315	88-100	98-105	363-410	353-405	438-475	453-553
	n	4	4	4	3	3	5	5	5	5	3	4	4	4	4
2N	M	549	375	213	227	88	225	204	249	77	61	331	318	373	391
	S	77	53	26	17	-	4	7	3	3	2	5	3	7	8
	R	500-665	330-450	191-250	205-241	-	220-230	198-213	225-260	73-80	60-63	328-338	315-323	365-383	380-398
	n	4	4	4	4	1	4	4	3	4	2	4	4	4	4
1N	M	508	375	168	325	-	171	162	210	65	30	265	n/a	305	288
	S	-	-	-	-	-	1	2	2	2	2	4	n/a	-	285-290
	R	-	-	-	-	-	170-172	160-163	206-213	64-65	25-30	263-265	n/a	-	4
	n	1	1	1	1	-	2	2	2	2	2	2	n/a	1	2
L	M	323	243	118	88	-	137	128	195	48	-	210	200	238	-

**Figure 3.**
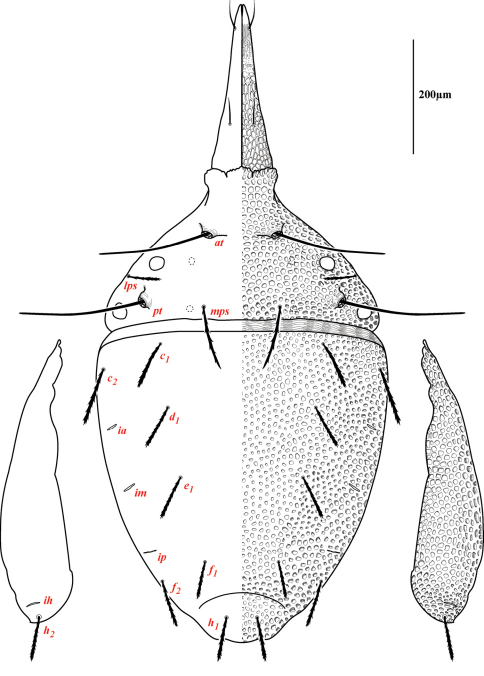
Dorsum of *Trachymolgus purpureus* sp. n. Lateral plates removed and displayed laterally. See text for abbreviations.

**Figure 4.**
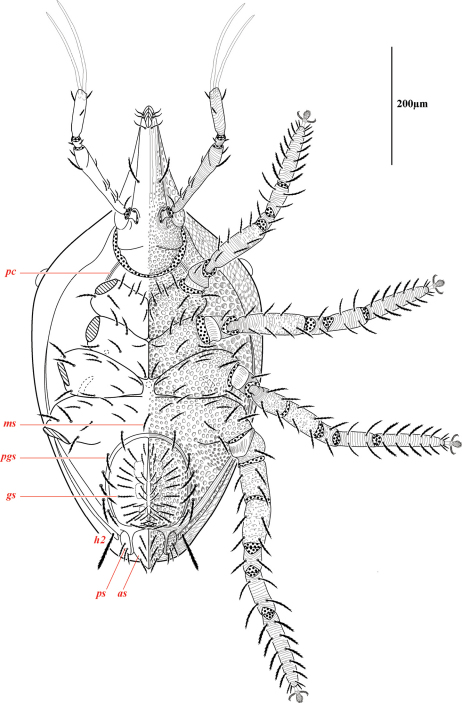
Venter of *Trachymolgus purpureus* sp. n. Podocephalic canal (*pc*), median seta (*ms*), paragenital setae (*pgs*), genital shield/setae (*gs*), paranal shield/setae (*ps*), anal shield/setae (*as*). Stippling denotes unstriated membrane.

**Figure 5.**
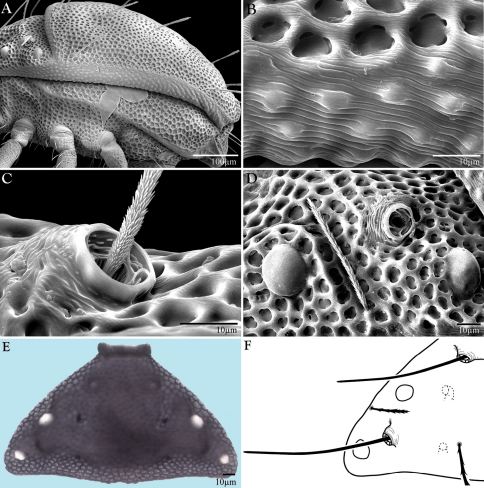
Morphological aspects of *Trachymolgus purpureus* sp. n. **A** LT-SEM of lateral view **B** enlargement of lateral membrane showing striations accompanied with bumps **C** Base of *pt* showing minute barbules **D** Left lateral view of *lps* in a groove above anterior eye, *pt* removed **E** Compound light micrograph of proterosomal shield with apodemes in focus, appearing as four dark spots **F** Line drawing of proterosomal shield showing apodemes.

Dorsal idiosoma(Fig. 3). **Idiosoma** dorsally armored with two large tergites: proterosomal and hysterosomal shields (see Terminology). Dorsal membrane (between proterosomal and hysterosomal shields and between dorsal and lateral shields) striated and accompanied with raised bumps similar in size to the foveolate indentions (Figs 4, 5a-b). **Proterosoma** ending anteriorly in a crenulated, tri-lobed shelf (crown) covering the stigmata. Two pairs of eyes present. Two pairs of minutely barbulate trichobothria: anterior (*at*) and posterior trichobothria (*pt*). Barbules are difficult to discern with light microscopy (Fig. 5c). Two pairs of barbulate setae are present: lateral proterosomal (*lps*) and median proterosomal setae (*mps*). Setae *lps* are oriented dorsomedially and lay in a groove posterior to the first pair of eyes (Fig. 5d); *mps* are the longest barbulate setae. Two pairs of heavily sclerotized, cylindrical, internally directed structures are apparent (Fig. 5e-f) that we interpret to be apodemes. **Hysterosoma** folding over posterior, shelf-like portion of proterosoma; with three lyrifissures (*ia*, *im*, and *ip*) and seven barbulate setae as follows: *c1*, *c2*, *d1*, *e1*, *f1*, *f2*, and *h1*. Posteriorly, the hysterosomal shield folds inward between the *f1-2* and *h1* forming a curved lateral furrow isolating *h1* on a raised area.

**Figure 6.**
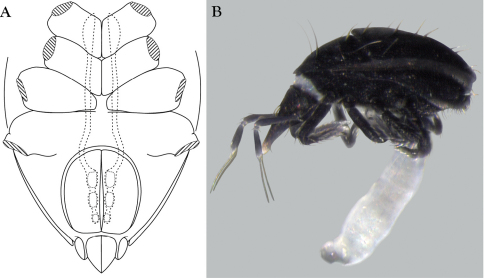
Ventral aspects of *Trachymolgus purpureus* sp. n. **A** Venter showing pseudotracheae, legs removed **B** Stereomicrograph showing extruded ovipositor.

**Figure 7.**
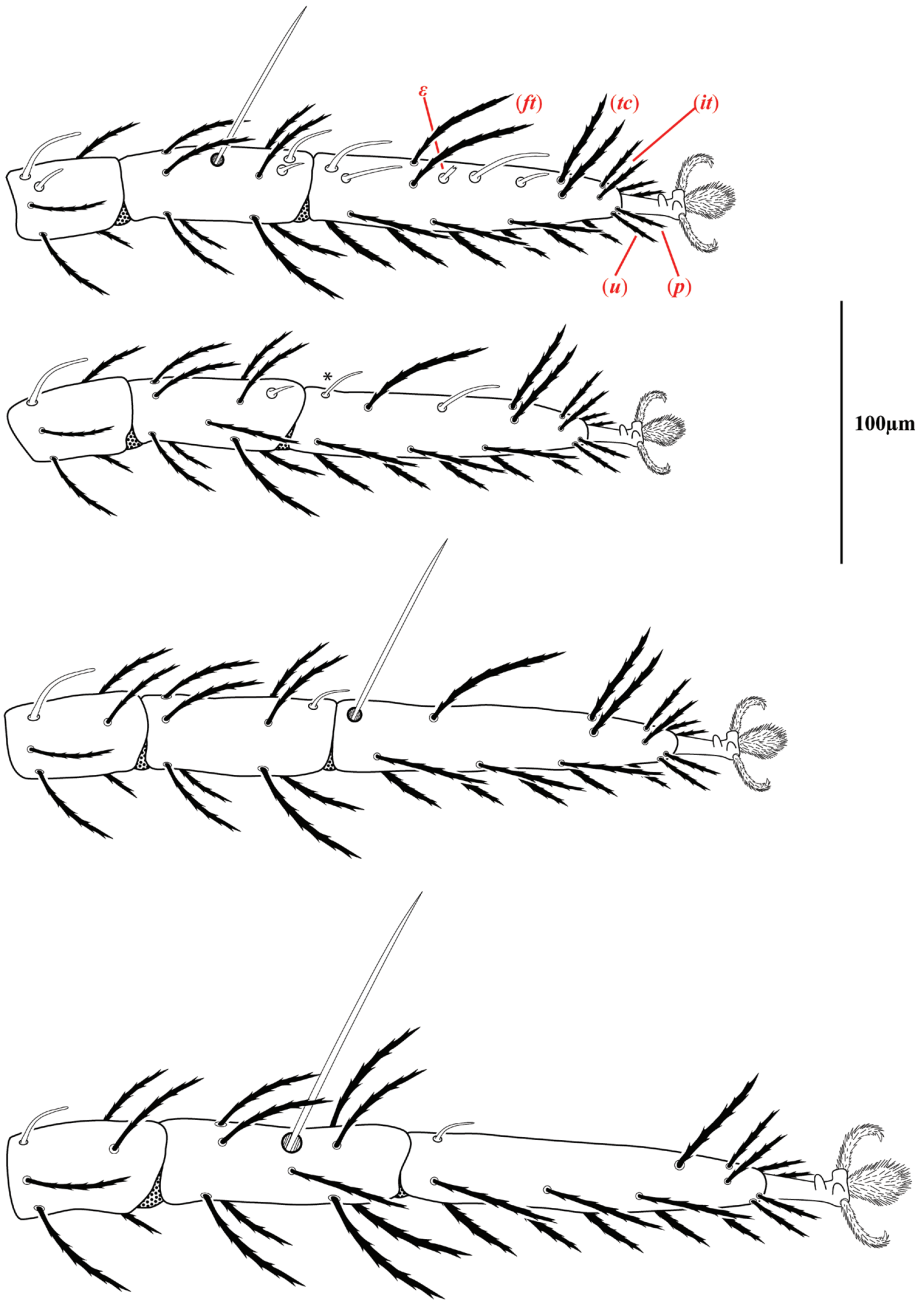
Legs of *Trachymolgus purpureus* sp. n. Laterodorsal view of distal podomeres. Fastigials (*ft*), iterals (*it*), prorals (*p*), tectals (*tc*), unguinals (*u*), and famulus (ε). Stippling denotes unstriated membrane. Asterisk (*) denotes solenidion found in only a few specimens.

**Figure 8.**
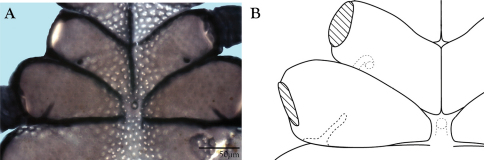
Coxal fields of *Trachymolgus purpureus* sp. n. **A** Compound light micrograph of venter showing apodemes on coxae II & III **B** Line drawing with emphasis on apodemes.

**Figure 9.**
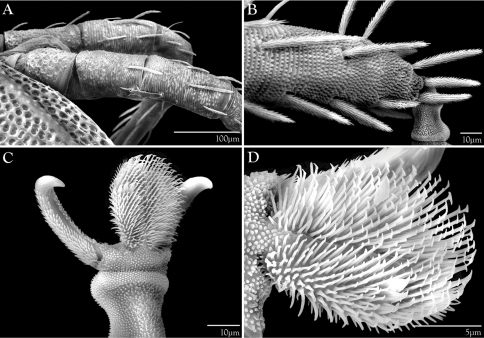
Legs of *Trachymolgus purpureus* sp. n. LT-SEM. **A** Leg II, showing sclerotized, pitted sculpturing on telofemur and genu **B** Tarsus I showing papillated striations **C** Apotele II showing barbulate ungues **D** Enlargement showing tenant hairs.

Ventral idiosoma(Fig. 4). **Lateral shields**each posteriorly containing one lyrifissure (*ih*) and one barbulate seta (*h*2). Podocephalic canals lead from the posteriolateral edges of the gnathosoma and curve around coxal field I, and are visible externally (Figs 1a, 4). Ventral membrane is striated but lacks bumps. **Genital region** covered with one pair of genital shields each containing more than 20 barbulate setae. There are six pairs of paragenital setae; one unpaired median seta between coxal field IV; three pairs of genital papillae; one pair of genital tracheae associated with the genital papillae that leads into the body anteriorly from the anterior-most genital papilla, and ending in spoon-shaped platytracheae near coxal field I (Fig. 6a). Female with long, telescoping ovipositor that approaches body length (Fig. 6b); with 16 setae. Male amphoid sclerites each with nine setae. Unpaired median cylindrical structure interpreted as an apodeme between coxal field III (Fig. 8). **Anal region** with two pairs of sclerites: anal shields and paranal shields, each usually containing three pairs of barbulate setae. Either side of both anal and paranal shields may have one to two extra setae (symmetrically or asymmetrically). **Legs**(Figs 4, 7): coxal fields I-III distinct, coxal field IV indistinguishably fused medially with venter; sclerotized, inwardly directed cylindrical structures (interpreted here as apodemes) are readily apparent on coxal field II and III (Fig. 8). Trochanters, femora, and genua sclerotized, with pitted, sculptured armor, especially II and III (Figs 4, 9a); other podomeres unsclerotized with papillated striations (Fig. 9b). Podomeres with eight possible setal rows positioned ventrally (unpaired), medioventrally (paired), lateroventrally (paired), laterally (paired), laterodorsally (paired), and dorsally (unpaired). Base of the ambulacrum surrounded with two pairs of setae: prorals (*p*) and unguinals (*u*). Proximally, the dorsal setae are as follows: iterals (*it*), tectals (*tc*), and fastigials (*ft*). The tectals are paired on all legs except IV; fastidials are paired only on leg I, and are absent on leg IV. Other setal homologies remain to be investigated. Baculiform solenidia present on genua I-IV (σ), tibiae I and III (γ), and tarsi I, II, and IV (ω); short, ceratiform solenidia present on tibiae I and II (γ); and a short solenidion present on tarsi I that has the appearance of being raggedly broken, interpreted here as the famulus (ε). Trichobothria present on tibiae I and IV, and tarsus III. Apotele with barbulate ungues and pulvilli with tenant hairs (Fig. 9c-d). Leg arthrodial membrane is unsculptured.

**Figure 10.**
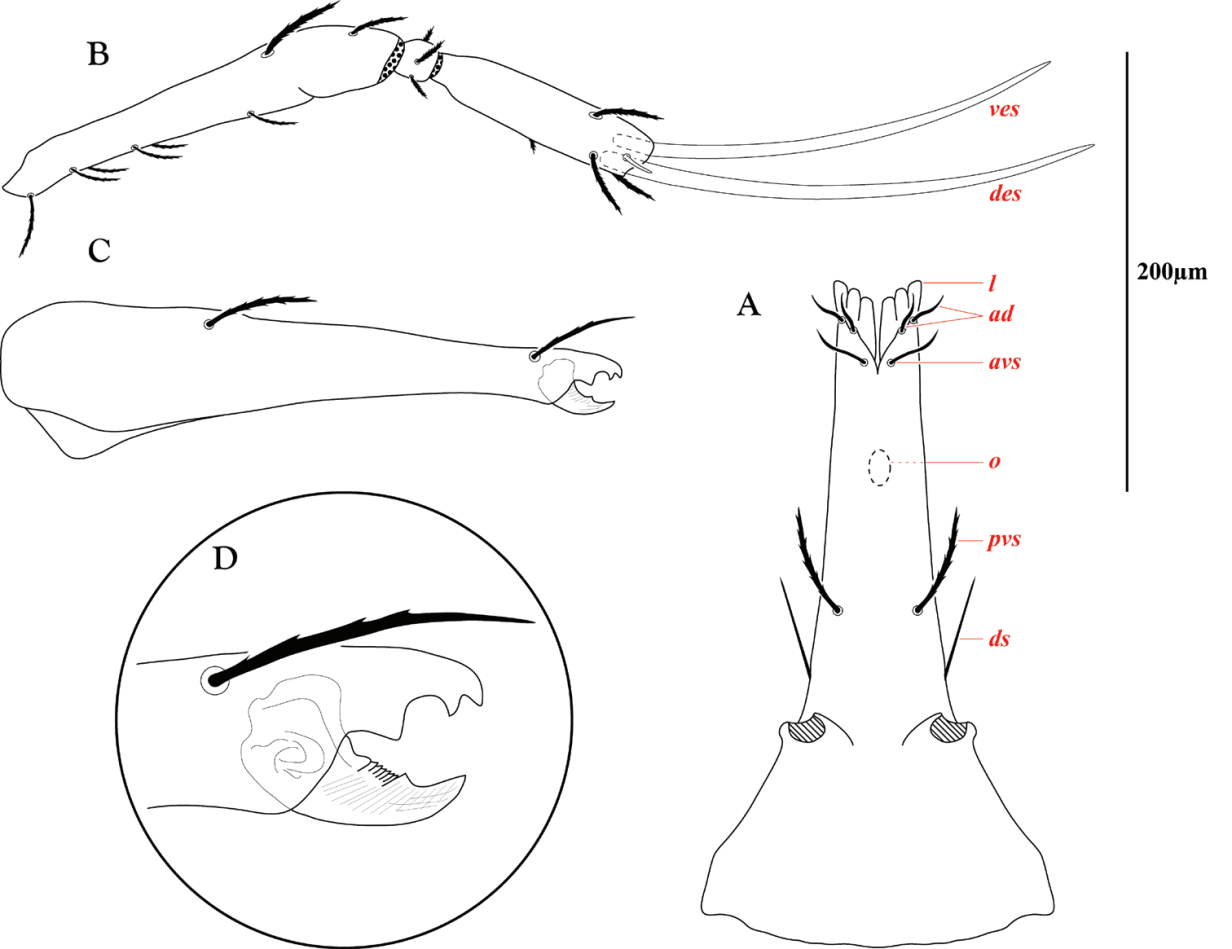
Gnathosoma of *Trachymolgus purpureus* sp. n. **A** Subcapitulum **B** Pedipalp **C** Chelicera **D** Chela enlarged. Ventral end seta (*ves*), dorsal end seta (*des*), lateral lips (*l*), adorals (*ad*), anterioventral subcapitular setae (a*vs*), oral opening (*o*), posterioventral subcapitular setae (p*vs*), dorsal subcapitular setae (*ds*). Stippling denotes unstriated membrane.

**Figure 11.**
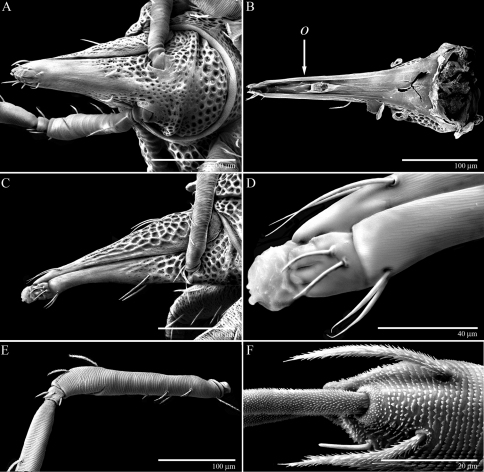
Gnathosoma of *Trachymolgus purpureus* sp. n. LT-SEM. **A** Ventral view of gnathosoma showing subcapitular sculpturing **B** Dorsal view of subcapitulum showing position of oral opening (*o*) **C** Lateral view of gnathosoma showing cheliceral sculpturing **D** Magnified view of distal gnathosoma showing lateral lips and silk charge **E** Dorsolateral view of removed pedipalp showing striations **F** Ventrodistal view of right pedipalp showing papillated striations, finely barbulate *ves*, and solenidion.

Gnathosoma(Fig. 10). **Subcapitulum** (Fig. 10a) foveolate and armored posteriorly, longitudinally striated anteriorly (Fig. 11a); ventrally with two pairs of smooth adoral setae (*ad*), one pair of smooth anterior setae (*avs*), and one pair of barbulate posterior setae (*pvs*); dorsally with one pair of smooth, thin, straight setae (*ds*) that are hidden under the chelicerae in life; ending in three pairs of lateral lips (Figs 10a, 11d). Oral opening located midway between ventral setae (Fig. 11b). Gnathosomal membrane unsculptured. **Pedipalps** (Fig. 10b) entirely striated (Fig. 11e), becoming more papillated-striated distally (Fig. 11f); femora partially fused dorsally; terminal setae (*ves* and *des*) finely barbulate (Fig. 11f). **Chelicerae** (Fig. 10c) with foveolate armoring basally, and longitudinal striation distally (Fig. 11c); with two dorsal barbulate setae. Fixed digit ending in a hook, and with two teeth (one small and one large and triangular); movable digit with one small tooth and a serrated edge proximal to the tooth (Fig. 10d).

**Table 3.** Dorsal setal measurements. Female (♀), male (♂), tritonymph (3N), deutonymph (2N), protonymph (1N), and larva (L), mean (M), standard deviation (S), range (R), number examined (n), anterior and posterior trichobothria (*at* and *pt*), lateral and medial proterosomal setae (*lps* and *mps*). All measurements in micrometers.

Stage		at	lps	pt	mps	c1	c2	d1	e1	f1	f2	h1	h2
♀	M	182	56	214	103	74	90	81	75	70	82	67	69
	S	23	11	18	8	6	17	8	3	5	12	3	4
	R	163-208	40-75	200-238	93-113	65-83	60-115	70-90	73-78	65-75	75-105	63-70	63-75
	n	3	7	4	7	5	7	4	4	3	6	5	7
♂	M	186	64	214	98	76	83	77	76	66	69	65	68
	S	4	7	15	4	1	8	7	5	11	8	7	5
	R	180-190	58-75	200-230	93-100	75-78	78-95	65-85	70-83	55-78	58-78	55-78	63-75
	n	4	6	3	5	3	4	5	4	3	6	6	6
3N	M	166	53	174	74	56	66	53	53	53	63	51	56
	S	18	4	2	3	6	6	1	2	4	3	1	7
	R	155-188	48-58	173-175	70-78	50-65	60-73	53-55	50-55	48-55	60-65	50-53	48-65
	n	3	5	2	4	5	4	4	4	4	4	4	4
2N	M	136	39	183	63	41	46	38	40	44	60	47	47
	S	2	1	-	3	4	4	0	2	4	2	2	3
	R	135-138	38-40	175-190	60-65	38-45	40-50	38	38-43	40-50	58-63	45-50	43-50
	n	2	4	1	3	3	4	4	4	4	4	4	4
1N	M	111	21	155	48	30	38	30	35	43	51	36	47
	S	34	8	49	-	-	4	-	-	3	1	1	1
	R	88-135	15-26	120-190	-	-	35-41	-	-	41-45	50-52	35-37	46-48
	n	2	2	2	1	1	2	1	1	2	2	2	2
L	M	105	20	118	43	30	33	33	40	40	43	40	40

**Table 4.** Gnathosomal measurements. Female (♀), male (♂), tritonymph (3N), deutonymph (2N), protonymph (1N), and larva (L), mean (M), standard deviation (S), range (R), number examined (n), dorsal subcapitulars (*ds*), proximoventral subcapitulars (*pvs*), distoventral subcapitulars (*dvs*), adorals (*ad*), dorsal end setae (*des*), ventral end setae (*ves*), cheliceral distal seta (*cds*), and cheliceral proximal seta (*cps*). All measurements in micrometers.

Stage		ds	pvs	dvs	ad	des	ves	cds	cps
♀	M	46	52	26	19	196	180	48	51
	S	4	6	2	2	10	22	5	5
	R	43-53	43-63	23-28	15-23	175-203	130-193	40-53	43-55
	n	5	7	7	8	7	7	8	7
♂	M	41	44	25	15	199	182	44	46
	S	7	6	4	4	16	9	3	6
	R	33-48	38-50	18-28	10-20	185-225	173-193	40-48	40-55
	n	4	3	5	5	5	5	6	5
3N	M	37	41	27	16	168	158	38	36
	S	1	2	4	3	8	5	1	5
	R	35-38	38-43	20-30	13-20	160-180	153-165	38-40	30-40
	n	4	5	5	5	5	5	3	3
2N	M	31	36	21	14	143	133	37	31
	S	2	2	3	1	3	3	3	1
	R	28-33	33-38	20-25	13-15	140-145	130-135	33-40	30-33
	n	4	4	4	4	3	3	4	4
1N	M	20	30	20	13	121	115	29	32
	S	-	-	-	-	118-125	113-118	-	-
	R	-	-	-	-	5	4	-	-
	n	1	1	1	1	2	2	1	1
L	M	19	30	24	12	100	85	30	34

##### Immatures description.

**Figure 12.**
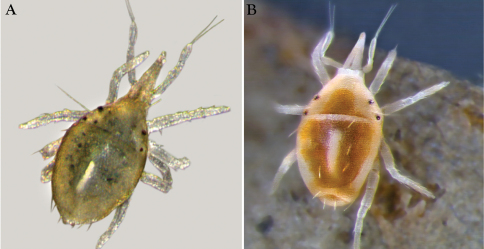
Nymphs of *Trachymolgus purpureus* sp. n.**A** Stereomicrograph showing greenish nymph, deutonymph shown **B** Stereomicrograph showing yellowish-white nymph,

**Figure 13.**
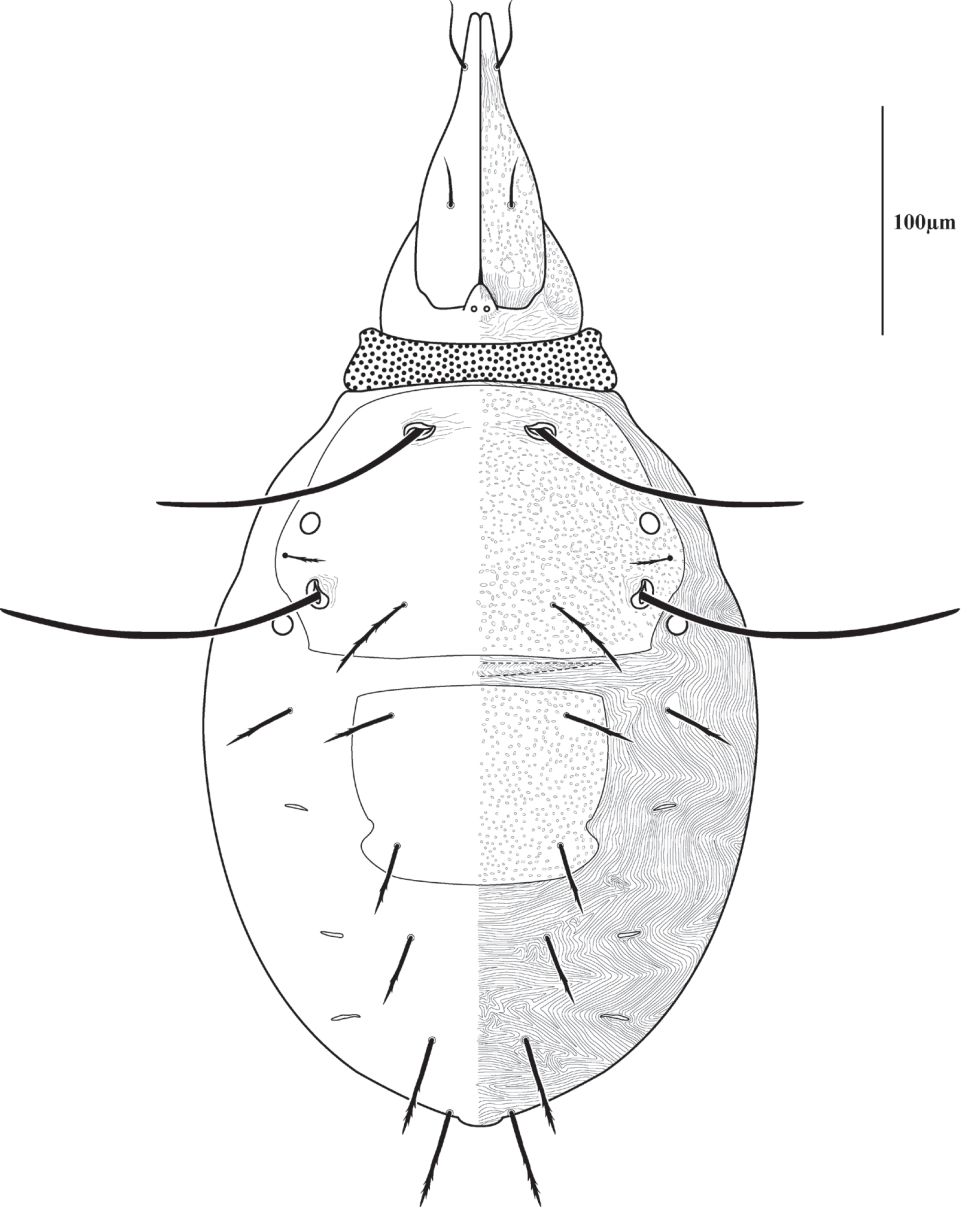
Larva of *Trachymolgus purpureus* sp. n. See dorsal illustration (Fig. 3) for labeling. Stippling denotes unstriated membrane. Note *f2* is lacking.

**Figure 14.**
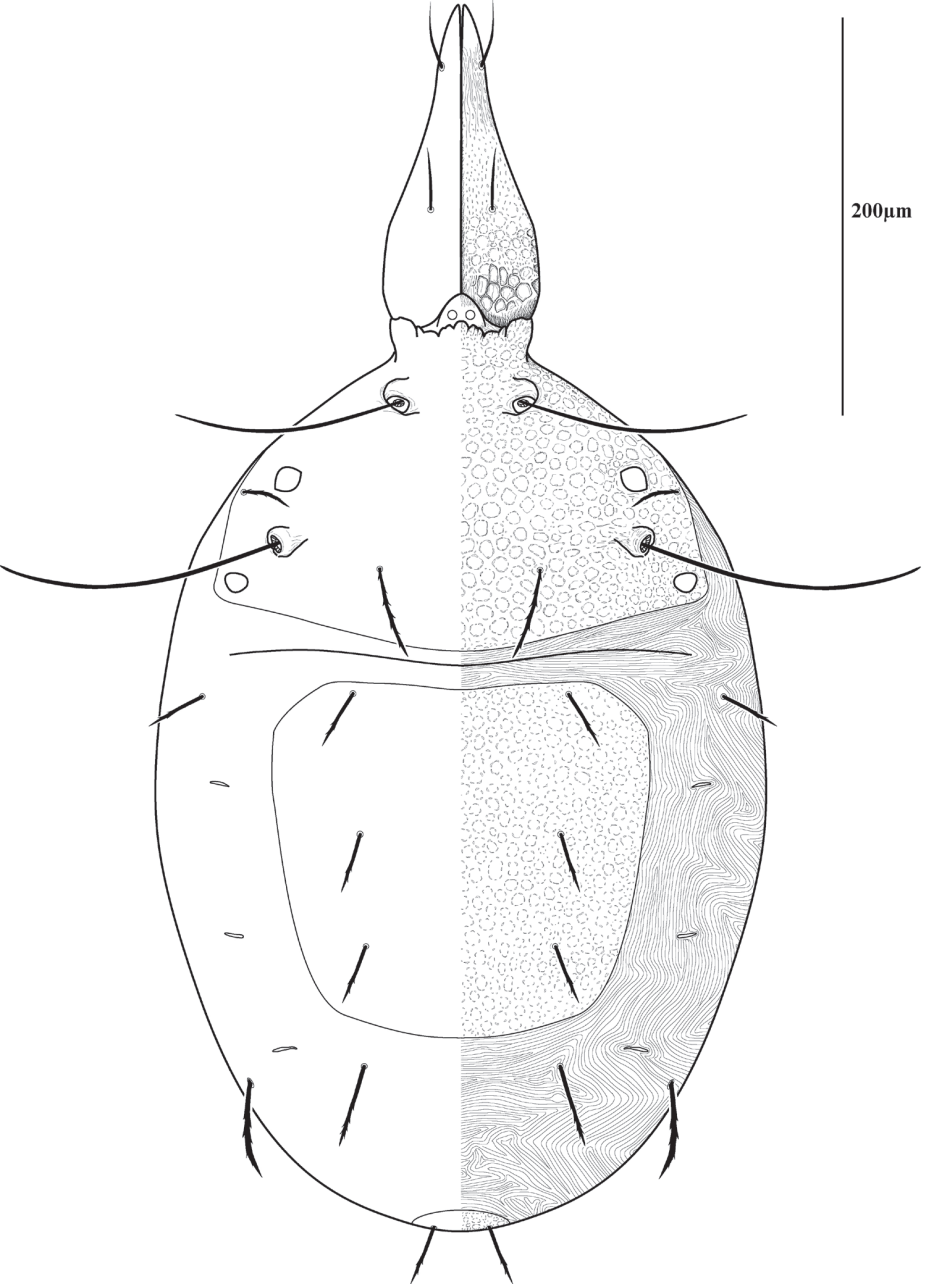
Protonymph of *Trachymolgus purpureus* sp. n. See Fig. 3 for labeling.

**Figure 15.**
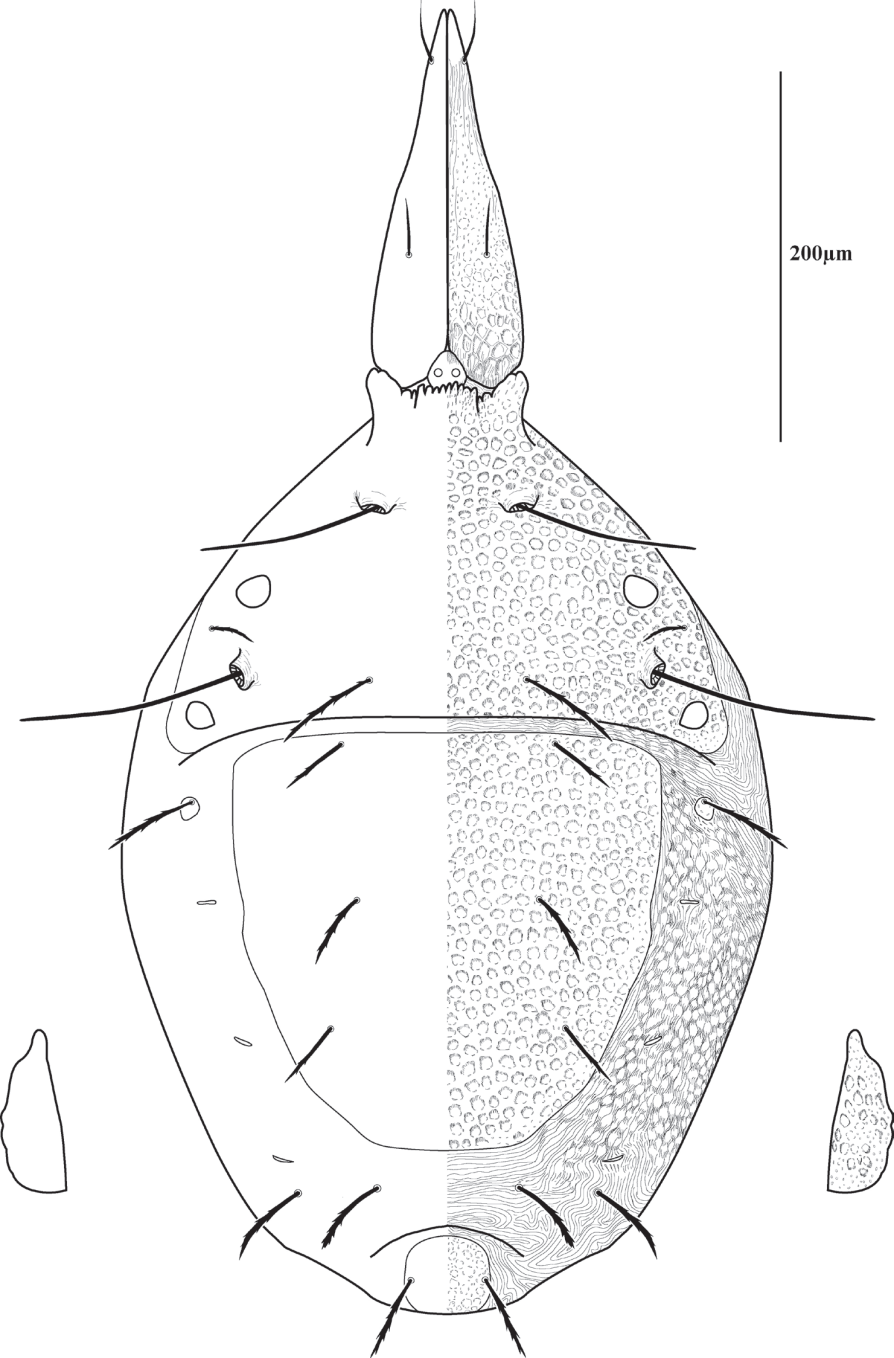
Deutonymph of *Trachymolgus purpureus* sp. n. Lateral plates removed and shown laterally. See Fig. 3 for labeling.

**Figure 16.**
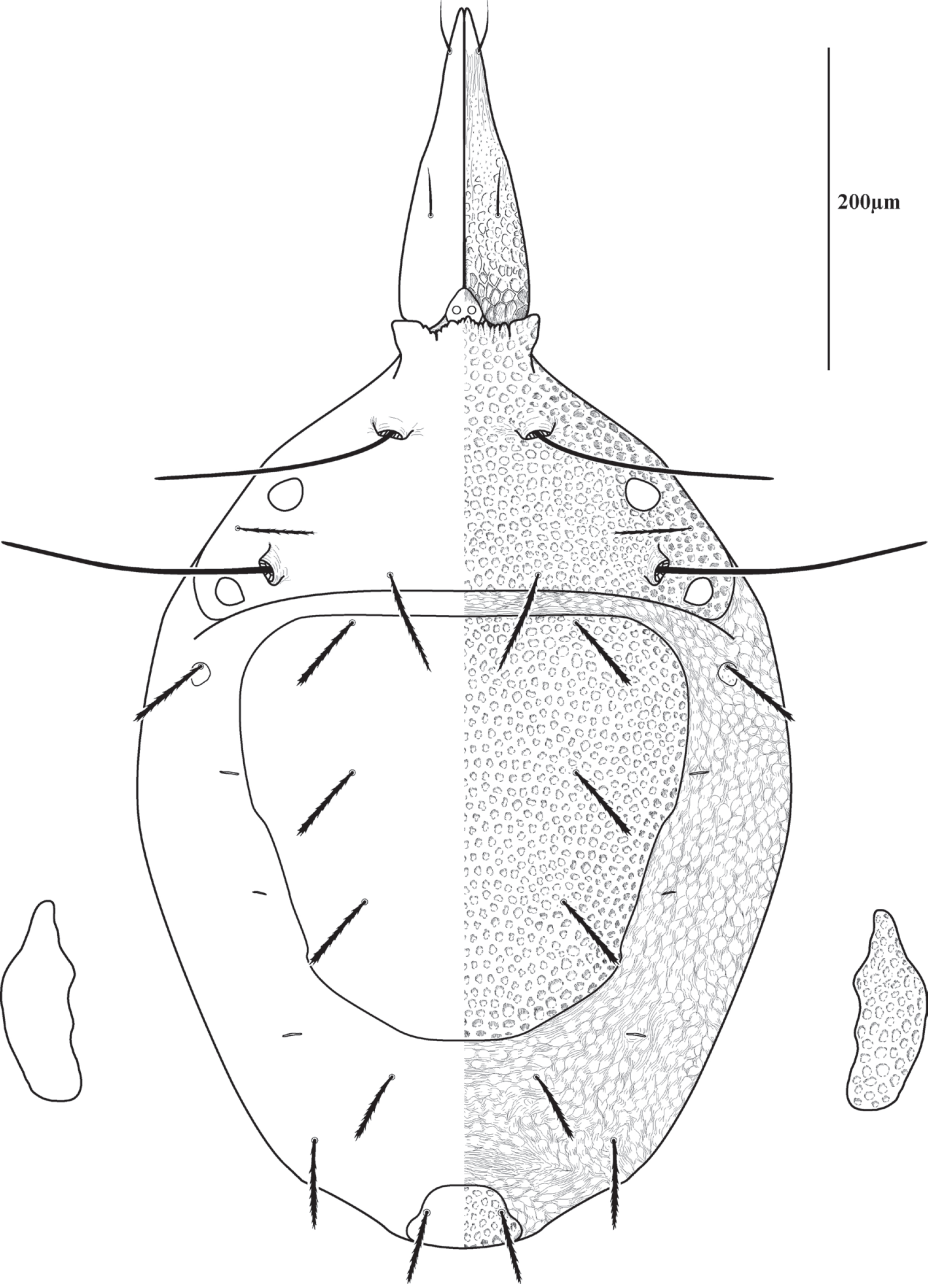
Tritonymph of *Trachymolgus purpureus* sp. n. Lateral plates removed and shown laterally. See Fig. 3 for labeling.

**Figure 17.**
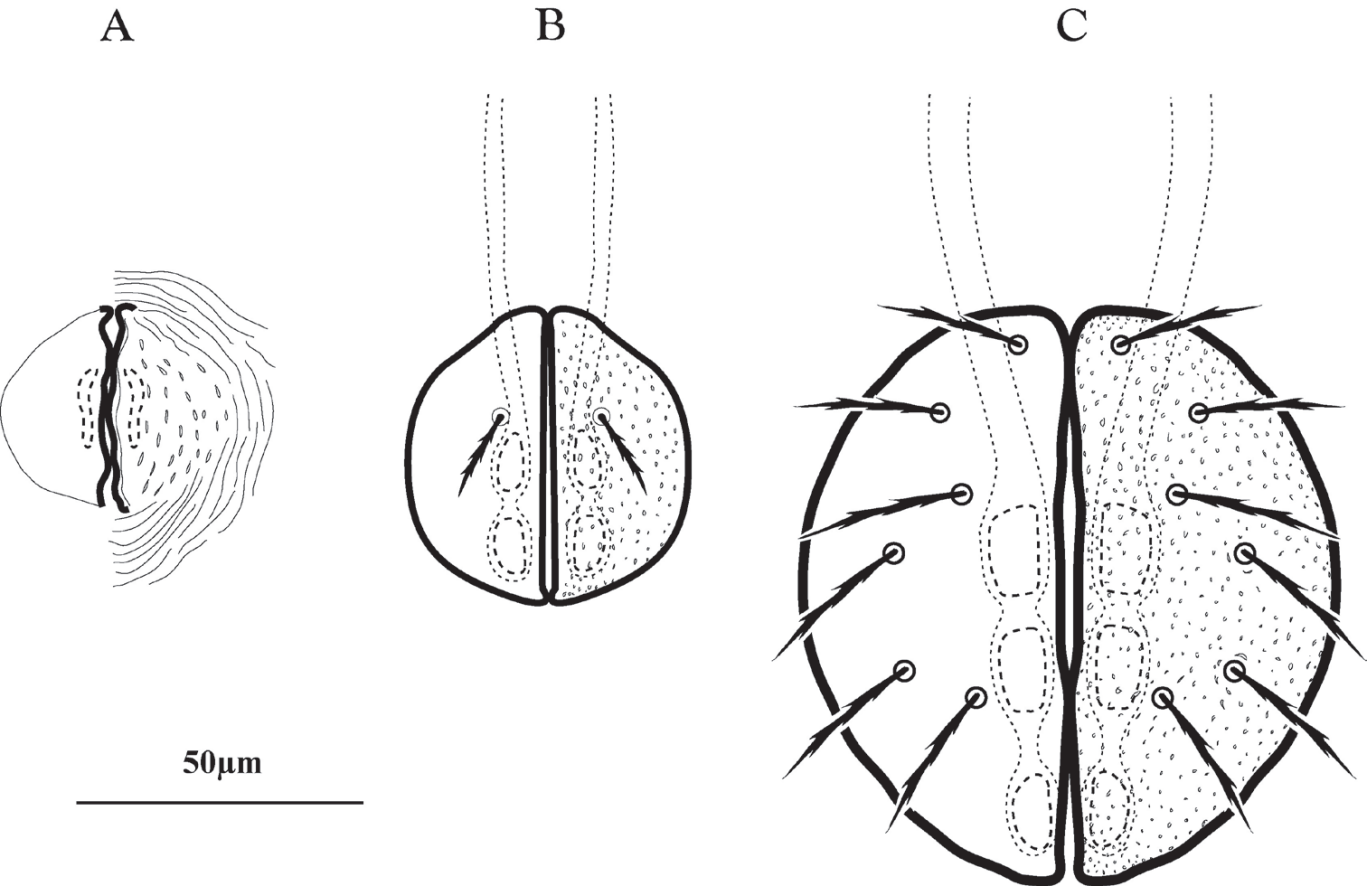
Genital development of immature *Trachymolgus purpureus*. **A** Protonymph (note weak sclerotization) **B** Deutonymph **C** Tritonymph.

Measurements and chaetotaxy of immatures are given in Tables 1–6. Developmental stages are illustrated in Figures 13–16. Like other mites, developmental stages can be easily recognized by leg number (larvae have six legs) and genital development (Fig. 17). Chaetotaxic differences and femoral divisions are also helpful (Tables 1, 6). All immature stages appear soft bodied (despite dorsal sclerites) and vary in color from light green or purple to yellowish-white (Fig. 12).

Due to the unique armored morphology of *Trachymolgus*, other interesting developmental changes are present. These are discussed below.

Dorsal sclerites and setae.None of the immature stages of *Trachymolgus purpureus* have complete dorsal shields as seen in the adult. This is unlike *Trachymolgus jesusi*, which was described as having an armored tritonymph and unsclerotized proto- and deutonymphs. In *Trachymolgus purpureus*, all stages have dorsal sclerites. Shield sculpturingis underdeveloped in the larva with foveolate indentions absent but pits present (Fig. 13); protonymphs also lack foveolate indentions, but the pits are more organized, reminiscent of the indentions (Fig. 14); deutonymphs begin to develop foveolate indentions (Fig. 15), which are nearly complete in the tritonymph (Fig. 16). The proterosomal shield of the larva does not encompass the posterior pair of eyes, and the anterior crown is not developed, leaving the gnathosomal membrane appearing as a collar. The protonymph has a well-developed proterosomal shield that encompasses all eyes and has a complete crown. Hysterosomal shield of the larva only encompasses *c1* and *d1*; nymphal stages also encompass *e1*. Small sclerotized regions containing pits, but not foveolate indentions, are present around *c2* in the deuto- and tritonymphs. A posterior shield encompassing *h1*, contiguous with the hysterosomal shield in adults, is present in nymphal stages, but not larvae. Larvae completely lack *f2*.

Lateral shields.Lateral shields are present in deuto- and tritonymphs (Figs 15, 16), but do not encompass *h2* or *ih*, as in adults (Fig. 3). Furthermore, in addition to lateral shields, larvae lack *h2*. Lyrifissure *ih* was not found in any immature stage.

Pseudotracheae.As described for *Trachymolgus jesusi*, pseudotracheae are lacking in the larva and protonymph, but are well-developed in the deutonymph (Fig. 17).

Membranes.As discussed above, adult *Trachymolgus purpureus* striations are accompanied with bumps (Fig. 5b), unlike other bdellid membranes that exhibit fingerprint-like striations. However, larvae and protonymphs lack bumps and have typical fingerprint-like striations (Figs 13, 14). Membrane bumps begin to develop on the deutonymphal dorsum (Fig. 15), and are well developed in the tritonymph (Fig. 16). All stages have normal, fingerprint-like striations on the venter.

**Table 5.** Ventral setal measurements. Female (♀), male (♂), tritonymph (3N), deutonymph (2N), protonymph (1N), and larva (L), mean (M), range (R), number examined (n), anal setae (*as*), paranal setae (*ps*), genital setae (*gs*), paragenital setae (*ps*), unpaired median seta (*ums*). Absent characters are denoted with a dash (-). All measurements in micrometers.

Stage		as1	as2	as3	as4	as5	ps1	ps2	ps3	long gs	short gs	pgs	ums
♀	M	43	43	44	40	-	43	52	54	34	17	41	30
	S	1	2	3	-	-	4	3	2	2	2	2	1
	R	42-45	41-46	41-49	-	-	39-48	47-55	52-56	31-37	14-20	38-46	29-32
	n	6	6	5	1	-	6	5	5	7	6	7	4
♂	M	45	43	42	41	43	45	51	52	35	18	42	37
	S	4	7	2	3	-	3	1	6	5	4	6	3
	R	40-51	35-56	40-44	37-43	-	42-50	50-52	45-62	27-44	14-21	34-48	35-40
	n	6	6	5	4	1	6	5	6	6	3	6	3
3N	M	37	37	38	36	-	42	43	45	27	18	32	26
	S	2	3	3	-	-	2	4	4	2	2	2	1
	R	35-40	35-41	35-40	-	-	40-44	40-47	40-49	25-30	16-20	30-35	25-27
	n	4	4	3	1	-	4	4	4	4	3	4	3
2N	M	30	29	29	32	-	35	39	38	21	-	23	28
	S	5	1	2	1	-	2	3	2	2	-	3	3
	R	24-35	28-30	26-30	31-33	-	32-36	34-42	35-40	19-23	-	21-25	25-30
	n	4	4	4	2	-	4	4	4	2	-	2	3
1N	M	24	25	22	-	-	27	32	37	24	19	24	25
	S	0	1	2	-	-	4	3	4	-	-	-	-
	R	24	24-25	20-23	-	-	24-30	30-34	34-40	-	-	-	-
	n	2	2	2	-	-	2	2	2	1	1	1	1
L	M	20	21	23	-	-	-	-	-	-	-	-	-

**Table 6.** Recognizing life stages. Female (♀), male (♂), tritonymph (3N), deutonymph (2N), protonymph (1N), and larva (L). Numbers represent setal counts; those in parentheses denote counts when extra setae are present. Absent characters are denoted with a dash (-).

Stage	Adoral Setae	Anal Setae	Paranal Setae	Genital Setae	Paragenital Setae	Femora III divided	Femora IV divided
♀	2	3 (4)	3	>20	6	yes	yes
♂	2	3 (5)	3 (4)	>20	6	yes	yes
3N	2	3 (4)	3	6	5	yes	yes
2N	2	3 (4)	3	1	5	yes	yes
1N	2	3	3	0	0	yes	no
L	1	3	1	-	-	no	no

#### Remarks.

In the early 1980s, *Trachymolgus* was collected by Cal Welbourn on a rocky bluff in the Buffalo National River (Arkansas). John Kethley recollected three specimens from the same bluff a few years later. Another specimen (one female) was collected by Evert Lindquist in the St. Lawrence Islands National Park, Canada (Smith et al. 1996), but the specimen has since been lost. Since then, we have collected this seemingly restricted, rare mite from a wide variety of microhabitats including litter, talus, rock outcrops and bluffs, moss, cedar stands, hardwood stands, and in both wet and dry conditions. Furthermore, the range is potentially not restricted to the Ozark highlands. Amusingly, we collected one tritonymph and one adult from leaf litter less than 200ft from where the Ohio State University Summer Acarology Course is taught (downtown Columbus, Ohio). Morphologically, these specimens cannot yet be distinguished from *Trachymolgus purpureus*, potentially extending the range to eastern U.S. We were able to extract DNA from one of these specimens and will publish our findings later.

#### Biogeography.

The known distribution of North American *Trachymolgus* is Mexico (*Trachymolgus jesusi*), Ozark highlands (*Trachymolgus purpureus*), central Ohio (undet. species), and the northern Appalachian mountains (undet. species). Other groups have a similar distribution, and the biogeographic affinity between the Ozark and Appalachian mountains, and between Mexico and the eastern U.S. has been well documented. Examples include mosses (Crum 1952; Redfearn 1986), higher plants (Braun 1955; Dressler 1954; Miranda and Sharp 1950; Watson 1891), fungi (Miranda and Sharp 1950; Sharp 1948), snakes, flying squirrels, and plethodontid salamanders (see Martin and Harrel 1957). Recently, a mite was implicated as sharing this affinity (Skvarla et al. 2011). It is tempting consider *Trachymolgus purpureus* as representative of these biogeographic events, but much more sampling is necessary before this conclusion can be justified.

#### Temperature tolerance.

**Figure 18.**
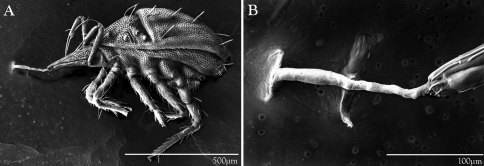
LT-SEM of silk production in *Trachymolgus purpureus* sp. n. **A** Lateral habitus showing frozen mite with legs curled, attached to LT-SEM plate with silk tether **B** Enlargement of anterior gnathosoma and silk tether.

*Trachymolgus purpureus* seems to have extremely high temperature tolerances. They were found crawling on rock surfaces in direct sunlight during a drought in the hottest and driest time of year (August), and were collected near the surface during the winter. When preparing live specimens for LT-SEM, mites are set atop a metal bar that is subjected to liquid nitrogen fumes which freezes them mid-stride for imaging. When *Trachymolgus purpureus* was subjected to liquid nitrogen temperatures however, they would simply run, curl their legs, and roll off the plate (see Fig. 18a). This made imaging live specimens very difficult.

#### Silk production.

LT-SEM imaging illuminated another behavioral characteristic of *Trachymolgus purpureus*. Though other bdellids have been known to orally produce silk to tether prey (Alberti 1973, Krantz and Walter 2009), silk production in *Trachymolgus* was not previously known. When subjected to liquid nitrogen, *Trachymolgus purpureus* would charge its gnathosoma with silk, making investigation of chelae impossible on living specimens (Fig. 11d). One specimen tethered itself to the plate before rolling off (Fig. 18), potentially using silk as a dragline as has been described in many spiders.

Feeding behavior.We observed a tritonymph of *Trachymolgus purpureus* feeding on a small mite approximately 200–250m long. Unfortunately, the prey could not be retrieved for identification. The tritonymph fed with prey elevated from the ground. There seemed to be a droplet surrounding the bite site, interpreted here as silk seen in Figures 11d. We hypothesize that *Trachymolgus purpureus* uses a drop of silk at the bite site to act as a gasket when sucking prey fluids.

#### Type material.

(27 individuals on slides). HOLOTYPE: female, collected from leaf litter, USA, Arkansas, Washington Co., Devil’s Den State Park (35°46.817 N, 94°14.750 W), 23 Sep 2009, by JR Fisher & MJ Skvarla, APGD 09-0923-006.

PARATYPES: **Female (n=7):** 2 individuals collected from leaf litter on rocky slope, USA, Arkansas, Washington Co., Devil’s Den State Park (35°46'50.1N, 94°14'45.9"W), 28 Aug 2008, by APG Dowling, APGD 08-0828-004 ● 2 individuals collected from leaf litter on rocky slope, USA, Arkansas, Washington Co. Devil’s Den State Park (35°46'50.1"N, 94°14'45.9"W), 30 Aug 2009 by JR Fisher, APGD 09-0830-001 ● 1 individual collected from leaf litter, USA, Arkansas, Newton Co., Buffalo National River, Roark Bluff (36°01'56.2"N, 93°20'01.5"W), 7 Sep 2009 by JR Fisher, APGD 09-0907-005 ● 1 individual collected from American beech leaf litter, USA, Arkansas, Newton Co., Buffalo National River, Boen Gulf (35°52.062 N, 093°24.092 W), 14 Mar 2010 by JR Fisher, APGD 10-0314-019 ● 1 individual collected from litter on rocky bluff, USA, Arkansas, Newton Co., Buffalo National River, Roark Bluff (36°01'56.2"N, 93°20'01.5"W), [date unknown] by Cal Welbourn, OSAL 0061853. **Male (n=5):** 1 individual collected from rocky overhang, USA, Arkansas, Washington Co., Devil’s Den State Park (35°46'50.1N, 94°14'45.9"W) by APG Dowling, APGD 08-0822-001 ● 1 individual collected from cedar litter, USA, Arkansas, Washington Co., Buffalo National River, Roark Bluff (36°01'56.2"N, 93°20'01.5"W) by JR Fisher, APGD 09-0802-006 ● 1 individual collected from leaf litter on rocky slope, USA, Arkansas, Washington Co. Devil’s Den State Park (35°46'50.1"N, 94°14'45.9"W), 30 Aug 2009 by JR Fisher, APGD 09-0830-001 ● 1 individual collected from leaf litter on rocky slope, USA, Arkansas, Washington Co. Devil’s Den State Park (35°46'50.1"N, 94°14'45.9"W), 30 Aug 2009 by JR Fisher, APGD 09-0830-003 ● 1 individual collected from leaf litter, USA, Arkansas, Newton Co., Buffalo National River, Roark Bluff (36°01'56.2"N, 93°20'01.5"W), 7 Sep 2009 by JR Fisher, APGD 09-0907-005. 1 individual collected from leaf litter, USA, Arkansas, Newton Co., Buffalo National River, Boen Gulf (35°52.062 N, 093°24.092 W), 10 Apr 2010 by APG Dowling. **Tritonymph(n=6):** 3 individuals collected from cedar litter, USA, Arkansas, Washington Co., Buffalo National River, Roark Bluff (36°01'56.2"N, 93°20'01.5"W) by JR Fisher, APGD 09-0802-006 ● 1 individual collected from oak litter, USA, Arkansas, Washington Co., Buffalo National River, Steel Creek trail (36°01'56.2"N, 93°20'01.5"W) by JR Fisher, APGD 09-0802-001 ● 1 individual collected from leaf litter, USA, Arkansas, Newton Co., Buffalo National River, Roark Bluff (36°01'56.2"N, 93°20'01.5"W), 7 Sep 2009 by JR Fisher, APGD 09-0907-005 ● 1 individual collected from litter on rocky bluff, USA, Arkansas, Newton Co., Buffalo National River, Roark Bluff (36°01'56.2"N, 93°20'01.5"W), [date unknown] by John Kethley, FMNH 2. **Deutonymph (n=4):** 3 individuals collected from cedar litter, USA, Arkansas, Washington Co., Buffalo National River, Roark Bluff (36°01'56.2"N, 93°20'01.5"W) by JR Fisher, APGD 09-0802-006. 1 individual collected from leaf litter on rocky slope, USA, Arkansas, Washington Co. Devil’s Den State Park (35°46'50.1"N, 94°14'45.9"W), 30 Aug 2009 by JR Fisher, APGD 09-0830-002. **Protonymph (n=2):** 1 individuals collected from leaf litter on rocky slope, USA, Arkansas, Washington Co., Devil’s Den State Park (35°46'50.1N, 94°14'45.9"W), 28 Aug 2008, by JR Fisher & MJ Skvarla, APGD 08-0828-004 ● 1 individual collected from cedar litter, USA, Arkansas, Washington Co., Buffalo National River, Roark Bluff (36°01'56.2"N, 93°20'01.5"W) by JR Fisher, APGD 09-0802-006. **Larva (n=1):** 1 individual collected from oak litter, USA, Arkansas, Washington Co., Buffalo National River, Steel Creek trail (36°01'56.2"N, 93°20'01.5"W) by JR Fisher, APGD 09-0802-001.

#### Etymology.

This species is named for the Latin "purpureus," meaning purple.

### Notes on *Trachymolgus recki* Gomelauri, 1961

*Trachymolgus recki* was described from two specimens from Georgia (Gomelauri 1961). Unlike other *Trachymolgus*, the integument of *Trachymolgus recki* was described as unarmored (despite having foveolate indentations and proterosomal crown) and yellowish. There was one tooth on the fixed digit, one pair of eyes, and the pedipalps were inconclusively described as “nearly fused” though they were completely fused in the illustration (Gomelauri 1961).There were 6–7 genital setae, and coxal field setae 3-4-4-5.

We have found that purple *Trachymolgus purpureus* immatures lose color more readily than adults when slide-mounted, and some immatures are yellowish-white in life (Fig. 12b). Also, though deuto- and tritonymphal shields are foveolate, they are not as heavily sclerotized as in adults, which could give the appearance of being un-armored. Adults of *Trachymolgus purpureus* and *Trachymolgus jesusi* have higher setal counts with >20 and 10 genital setae, respectively, and coxal field setae 7-7-9-8 (female *Trachymolgus purpureus*), 6-6-6-10 (male *Trachymolgus purpureus*), and 8-5-11-10 (*Trachymolgus jesusi*). Larvae, proto- and deutonymphs of *Trachymolgus jesusi* were described as having highly reduced eyes. Gomelauri observed only one pair eyes in *Trachymolgus recki*. This offers significant evidence to suggest the specimens used to describe *Trachymolgus recki* were immature. Since these specimens were said to have three pairs of genital papillae, we suggest the description of *Trachymolgus recki* was based on tritonymphs. Therefore, *Trachymolgus recki* is excluded from the key below.

### Notes on *Trachymolgus jesusi*, Mejia-Recamier & Palacios-Vargas, 1999

Aspects of the morphology and development described for *Trachymolgus jesusi* (Mejia-Recamier & Palacios-Vargas 1999), suggest major deviations from what is known from other Bdellidae. Unfortunately, we were unable to obtain type specimens of this species.

### Key to adult *Trachymolgus* Berlese (excluding *Trachymolgus recki*, likely a tritonymph – see above)

**Table:** 

1	Movable digit with 3 teeth; pedipalp basi- and telofemur completely fused; leg basi- and telofemur III-IV completely fused; dark purple; Mexico	*Trachymolgus jesusi*
–	Movable digit with 1 tooth; pedipalp basi- and telofemur either divided or only partially fused dorsally; leg basi- and telofemur divided; dark purple to black	2
2	Pedipalp basi- and telofemur divided; black; Palaearctic	*Trachymolgus nigerrimus*
–	Pedipalp basi- and telofemur fused dorsally; dark purple; U.S.A.	*Trachymolgus purpureus*

## Supplementary Material

XML Treatment for Trachymolgus purpureus
